# Effect of percutaneous acupoint electrical stimulation combined with nutritional support on gastrointestinal function and nutritional status of patients after unicondylar knee arthroplasty

**DOI:** 10.3389/fnut.2026.1705575

**Published:** 2026-05-29

**Authors:** Jia Liu, Xin Wang, Qingpeng Fang, Yijie Yang, Pengpeng Wang, Yunchao Zhao

**Affiliations:** 1 Department of Cardiology, The Third Xiangya Hospital, Central South University, Changsha, Hunan, China; 2 Department of Orthopedics, Cangzhou Hospital of Integrated TCM-WM, Cangzhou, Hebei, China; 3 Department of Anesthesiology, Cangzhou Hospital of Integrated TCM-WM, Cangzhou, Hebei, China; 4 Department of Nursing, Cangzhou Hospital of Integrated TCM-WM, Cangzhou, Hebei, China

**Keywords:** gastrointestinal function, nutrition support, orthopedics, percutaneous acupoint electrical stimulation, unicondylar knee arthroplasty

## Abstract

**Aim:**

This study aimed to investigate the effects of percutaneous acupoint electrical stimulation combined with nutritional support on the gastrointestinal function and nutritional status of patients following unicondylar knee arthroplasty (UKA).

**Methods:**

This study adopted a randomized controlled trial design. A total of 60 patients who underwent UKA at the Cangzhou Hospital of Integrated Traditional Chinese and Western Medicine, Hebei Province, from 1 January 2020 to 31 December 2022, were enrolled and randomly divided into the control group (CG, *n* = 30) and the experimental group (EG, *n* = 30). The CG received routine treatment and conventional nutritional support, while the EG received percutaneous acupoint electrical stimulation in addition to the same routine treatment and conventional nutritional support. The primary endpoints were the recovery time of bowel sounds, time to first flatus, and time to first defecation. The secondary endpoints included clinical efficacy; serum levels of motilin, gastrin, interleukin (IL)-2, and IL-6; Kolcaba Comfort Scale scores; incidence of adverse reactions; and nutritional indicators.

**Results:**

The total effective rate of the EG was 86.67%, which was significantly higher than that of the CG (63.33%) (*p* < 0.05). The recovery time of bowel sounds, time to first flatus, and time to first defecation in the EG were significantly shorter than those in the CG (*p* < 0.05). After treatment, the serum levels of motilin, gastrin, IL-2, and IL-6 decreased in both groups; the serum motilin and gastrin levels in the EG were higher than those in the CG (*p* < 0.05), while the serum IL-2 and IL-6 levels in the EG were lower than those in the CG (*p* < 0.05). After treatment, the Kolcaba Comfort Scale scores in both groups increased, and the EG score was higher than that of the CG (*p* < 0.05). The incidence of adverse reactions in the EG was lower than in the CG (*p* < 0.05). The serum levels of total protein (TP), albumin (ALB), and prealbumin (PAB) in the EG were higher than those in the CG (*p* < 0.05).

**Conclusion:**

Percutaneous acupoint electrical stimulation combined with nutritional support can effectively promote gastrointestinal function and improve the nutritional status of patients after UKA.

## Introduction

Knee osteoarthritis is highly prevalent among middle-aged and elderly individuals, exerting a significant negative impact on the health and well-being of elderly patients (1). For advanced cases of knee osteoarthritis, knee replacement surgery is the standard therapeutic approach, and unicondylar knee arthroplasty (UKA) is a primary treatment option for advanced anteromedial knee osteoarthritis. Notably, favorable therapeutic outcomes have been achieved with UKA even in elderly patients (2).

However, a prominent clinical challenge following UKA is the high incidence of postoperative abdominal distension and constipation (3). Postoperative gastrointestinal function is weakened, increasing the risk of malnutrition, electrolyte disturbances, and poor wound healing in patients, all of which hinder rapid postoperative recovery (4). Gastrointestinal dysfunction, a common postoperative complication, is primarily associated with anesthetic stimulation, surgical stress, changes in defecation patterns, and environmental alterations. While this issue has been extensively documented in patients undergoing abdominal surgery, the incidence of abdominal distension and constipation after UKA has rarely been reported in the literature, despite being frequently observed in clinical practice (5). Consequently, this postoperative gastrointestinal dysfunction following UKA deserves the attention of orthopedic clinicians (6).

Although the long-term postoperative outcomes of UKA are generally satisfactory, the presence of abdominal distension and constipation reduces patient satisfaction during hospitalization (7). These gastrointestinal complications not only impair the patient’s quality of life but also have adverse effects on their nutritional status. Benign orthopedic diseases such as knee osteoarthritis can limit the normal activities of elderly orthopedic patients, leading to a decline in digestive and absorptive function, deterioration of nutritional status, and an increased risk of mortality (8).

Currently, both domestic and international nutritional treatment guidelines recommend enteral nutrition as the first-line option for patients with viable gastrointestinal function, with pre-digested formulas preferred for those with poor digestive and absorptive capacity (9). However, pre-digested formulations may lack enteral nutrition preparations rich in branched-chain amino acids and short peptides. Oral hydrolyzed protein, a protein supplement abundant in essential amino acids and short peptides, can provide a nitrogen source and effectively improve the nitrogen balance of patients (10).

Given the clinical significance of postoperative gastrointestinal dysfunction and malnutrition following UKA, there is an urgent need for effective intervention measures. This study aimed to evaluate the application of percutaneous acupoint electrical stimulation combined with nutritional support during the postoperative anesthesia recovery period. By comparing postoperative efficacy between the two groups, this study seeks to provide a theoretical basis for the prevention and treatment of postoperative gastrointestinal dysfunction following UKA and to guide clinical treatment strategies.

## Data and methods

### Study design

This study was designed as a randomized controlled trial. A total of 60 patients who underwent UKA at the Cangzhou Hospital of Integrated Traditional Chinese and Western Medicine, Hebei Province, from 1 January 2020 to 31 December 2022, were enrolled. The study was approved by the Ethics Committee of Cangzhou Hospital of Integrated Traditional Chinese and Western Medicine, and all patients signed an informed consent form prior to participation.

### Sample size calculation

The sample size was estimated based on the primary outcome (time to first flatus) using data from a previous pilot study. Assuming a mean difference of 8 h between groups with a standard deviation of 10 h, a two-sided significance level of *α* = 0.05, and a statistical power of 80%, the required sample size was calculated to be 25 patients per group. Considering a 20% attrition rate, 30 patients per group were enrolled. This calculation ensures that the study is adequately powered to detect a clinically meaningful difference at the primary endpoint.

### Randomization and blinding

Patients were randomly divided into the control group (CG, *n* = 30) and the experimental group (EG, *n* = 30) using a random number table. First, all 60 eligible patients were assigned a unique serial number from 1 to 60. A series of random numbers was then generated using a random number table, and the first 30 patients corresponding to the random numbers were allocated to the CG, while the remaining 30 patients were assigned to the EG. Allocation concealment was implemented using sealed envelopes: each patient’s group assignment was written on a piece of paper, placed inside an opaque, sealed envelope, and opened sequentially when the patient was ready to be enrolled in the study.

Due to the nature of percutaneous acupoint electrical stimulation, patients were aware of their group assignments because they could directly perceive the electrical stimulation. To minimize bias in outcome assessment, the assessors responsible for collecting data on various outcomes were blinded to the group assignments. They followed standardized data collection protocols and were not informed of the treatment received by each patient. Additionally, the statisticians responsible for data analysis were blinded to group assignments; the data were coded so that they could not identify the group to which each data point belonged until the final analysis was completed, ensuring objective statistical analysis.

### Inclusion criteria and exclusion criteria

Inclusion criteria: (1) Patients who meet the surgical indications for UKA, (2) patients aged between 45 and 65 years, regardless of gender, and (3) patients with normal bowel function before surgery.

Exclusion criteria: (1) Patients with cardiac pacemaker implantation, (2) patients with preoperative constipation or diarrhea, and (3) patients who refused to sign the informed consent form or requested withdrawal from the study.

### Therapeutic regimen

#### Anesthesia method

Both groups received subarachnoid anesthesia.

#### Surgical method

All patients in both groups underwent UKA. After satisfactory anesthesia induction, patients were placed in a supine position on the operating table. Routine disinfection and sterile draping were performed, followed by an incision on the medial side of the knee joint. The skin and subcutaneous tissue were incised sequentially, the joint space of the knee was exposed, osteotomy was performed, and the prosthesis was implanted. Flexion–extension movements and joint stability were checked postoperatively. After verifying the completeness of surgical instruments, a cocktail analgesic was injected around the joint, and the incision was closed layer by layer.

#### Treatment during postoperative anesthesia recovery

The CG received routine postoperative care, including routine electrocardiogram monitoring, pulse oxygen saturation monitoring, oxygen inhalation, and continuous recording of vital signs. Patients were transferred back to the ward once their vital signs were confirmed to be stable and accurate.

On the basis of the same routine treatment as the CG, the EG additionally received percutaneous acupoint electrical stimulation. The selected acupoints—Hegu (LI4), Quchi (LI11), Tianshu (ST25), Taibai (SP3), and Zhongwan (CV12)—are commonly used in traditional Chinese medicine for regulating gastrointestinal function. Hegu and Quchi are known to modulate immune and inflammatory responses, while Tianshu, Taibai, and Zhongwan directly regulate gastric motility and intestinal transit. The electrical stimulation parameters were set as follows: frequency of 2 Hz and intensity ranging from 5 to 10 mA (initially set at 5 mA for all patients to ensure safety and tolerance, then gradually increased to 10 mA based on individual patient response and subjective feelings of comfort). A square-wave waveform was adopted, and the stimulation duration was 30 min per session.

### Nutrition support

Patients in the CG received conventional nutritional support, which adhered to general nutritional guidelines for postoperative patients without the use of specialized enteral or parenteral nutrition products. The dietary plan was formulated to provide a balanced and appropriate intake of nutrients tailored to individual patient needs. The daily calorie intake was adjusted based on each patient’s estimated energy requirements, with a target of approximately 20–25 kcal/kg/day for patients with normal metabolic rates. This caloric goal was achieved through a combination of grains, proteins, vegetables, and fruits. The daily protein target was 0.8–1.0 g/kg/day, with an emphasis on high-quality protein sources such as lean meat (e.g., chicken breast and beef tenderloin), fish (e.g., salmon and cod), eggs, dairy products (e.g., milk and yogurt), and legumes (e.g., lentils and chickpeas). Patients were allowed to resume normal oral intake on the second postoperative day, starting with a light and easily digestible diet (e.g., porridge, clear soups, and soft-boiled vegetables) and gradually transitioning to a normal diet as gastrointestinal function recovered, under the close monitoring of the medical team.

In addition to the same conventional nutritional support as the CG, patients in the EG were administered hydrolyzed protein oral solution (Xidakang, Kangzhe Hunan Pharmaceutical Co., Ltd.) at a dose of 50 mL per dose, twice daily. The administration time was between meals and before bedtime. Xidakang hydrolyzed protein oral solution mainly contains hydrolyzed whey protein, which is rich in essential amino acids and short-chain peptides. The nutritional support with hydrolyzed protein oral solution in the EG lasted for 5 days, starting on the second postoperative day (consistent with the time when both groups began normal oral intake).

### Primary endpoint

The primary endpoints of this study were the recovery time of bowel sounds, time to first flatus, and time to first defecation.

### Secondary endpoints

Clinical efficacy was evaluated in accordance with the Roman IV diagnostic criteria: Excellent: Bowel sounds recovered to 3–5 times/min, with defecation or flatus occurring within 1 day after surgery; Good: Bowel sounds recovered to 1–2 times/min, with defecation or flatus occurring within 2 days after surgery; Poor: No defecation, flatus, or bowel sounds observed within 2 days after surgery. The total effective rate was calculated as (number of excellent cases + number of good cases)/total number of cases × 100%.

Fasting venous blood (5 mL) was collected from each patient. The whole blood was centrifuged at 4,000 r/min at 4 °C for 15 min to separate the serum, which was then stored at −80 °C until detection. Serum levels of motilin, gastrin, IL-2, and IL-6 were determined by enzyme-linked immunosorbent assay (ELISA).

The Kolcaba Comfort Scale was used to evaluate patient comfort, which comprehensively assesses the physical, psychospiritual, environmental, and social dimensions of comfort. In the postoperative period of orthopedic surgeries such as UKA, patients experience not only physical pain but also psychological stress, and this scale can effectively capture these multifaceted aspects. Although specific validation for postoperative orthopedic populations is lacking, its wide application in various healthcare settings and proven reliability and validity in general patient groups support its use in this study. The scale score ranges from 28 to 112 points, with higher scores indicating higher levels of physical comfort.

The incidence of adverse reactions (nausea, vomiting, and abdominal distension) was recorded in both groups.

Nutritional indicators, including serum levels of TP, ALB, and PAB, were compared between the two groups 5 days after the initiation of nutritional support.

### Statistical analysis

SPSS 21.0 statistical software was used for data processing. Prior to statistical analysis, the Shapiro–Wilk test was performed to verify the normality of measurement data. Measurement data conforming to a normal distribution were expressed as (*x* ± *s*) and compared between groups using the independent-samples t-test. Measurement data not conforming to a normal distribution were compared using the Mann–Whitney *U*-test. Categorical data were expressed as *n* (%), and comparisons between groups were performed using the *χ*^2^ test. To enhance the clinical interpretability of the results, effect sizes were reported with 95% confidence intervals (CIs). Given the multiple comparisons conducted in this study, the risk of type I error was acknowledged. Although no Bonferroni correction was applied due to the exploratory nature of this study, this limitation was noted in the Discussion section, and strict adjustments will be implemented in future confirmatory studies. A *p*-value of < 0.05 was considered statistically significant.

## Results

### Baseline data between the two groups

The CG included 18 males and 12 females, aged 45–65 years, with a mean age of 58.64 ± 5.64 years. The EG included 17 males and 13 females, aged 46–64 years, with a mean age of 58.60 ± 5.58 years. No statistically significant differences were observed in age or gender between the two groups (*p* > 0.05), indicating that the baseline data were comparable.

### Clinical effect in both groups

As shown in Table 1, the total effective rate of the EG was 86.67%, which was significantly higher than that of the CG (63.33%) (*p* = 0.036, 95% CI: 0.368–0.963).

**Table 1:** Clinical effect between the two groups.

Groups	Cases	Excellent	Good	Bad	Total effective rate
Control group	30	10	9	11	19 (63.33%)
Experimental group	30	18	8	4	26 (86.67%)

### Recovery time of bowel sound recovery, first anal flatus, and defecation in both groups

**Figure 1:**
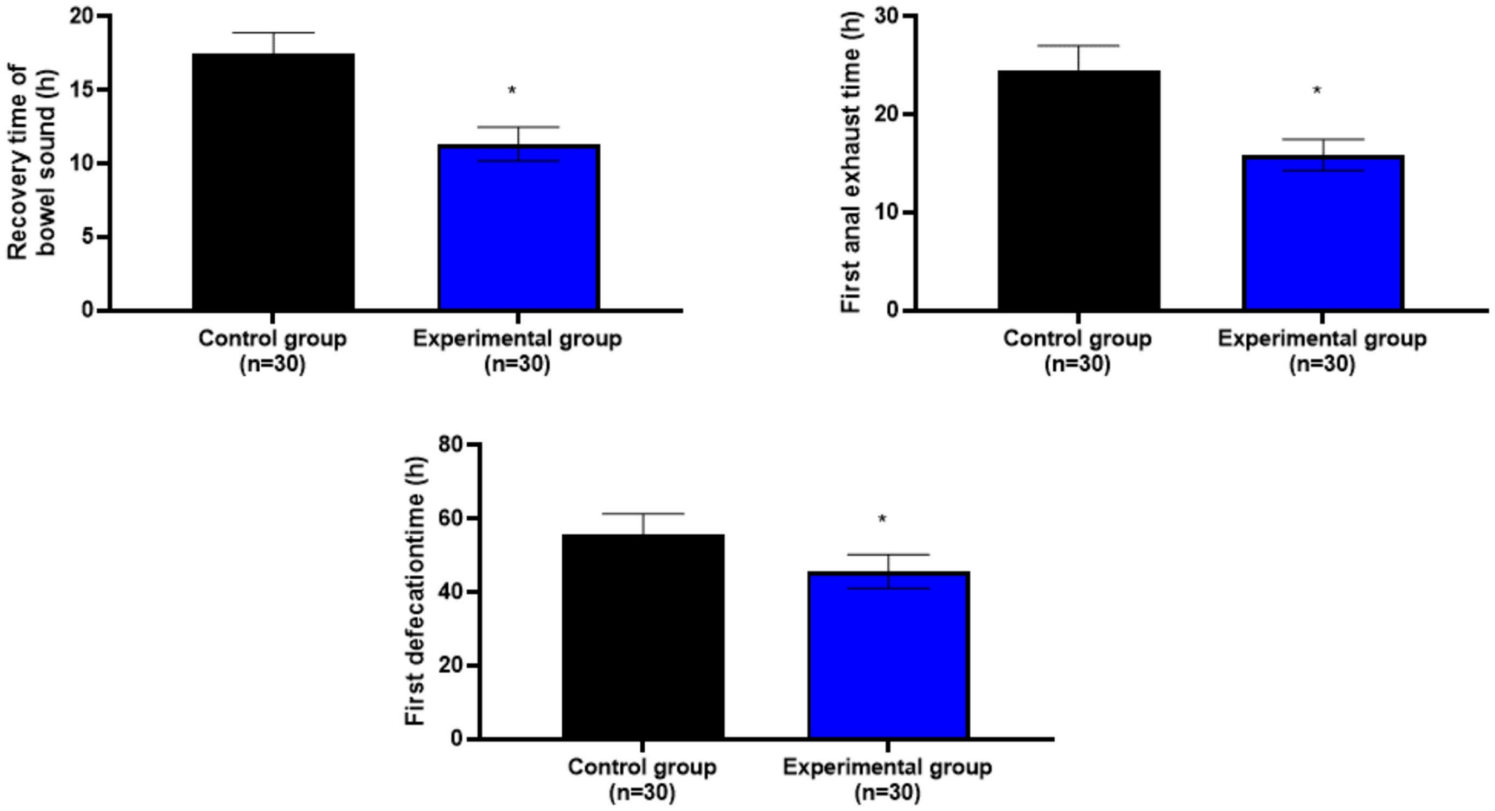
Recovery time of bowel sounds, first anal flatus, and defecation in both groups. ^*^p < 0.05.

As shown in Figure 1, the recovery time of bowel sounds, time to first anal flatus, and time to first defecation in the EG were significantly shorter than those in the CG (*p* < 0.05, 95% CI: −6.809 to −5.451; *p* < 0.05, 95% CI: −9.761 to −7.619; *p* < 0.05, 95% CI: −12.73 to −7.448).

### Laboratory indices in both groups

**Figure 2:**
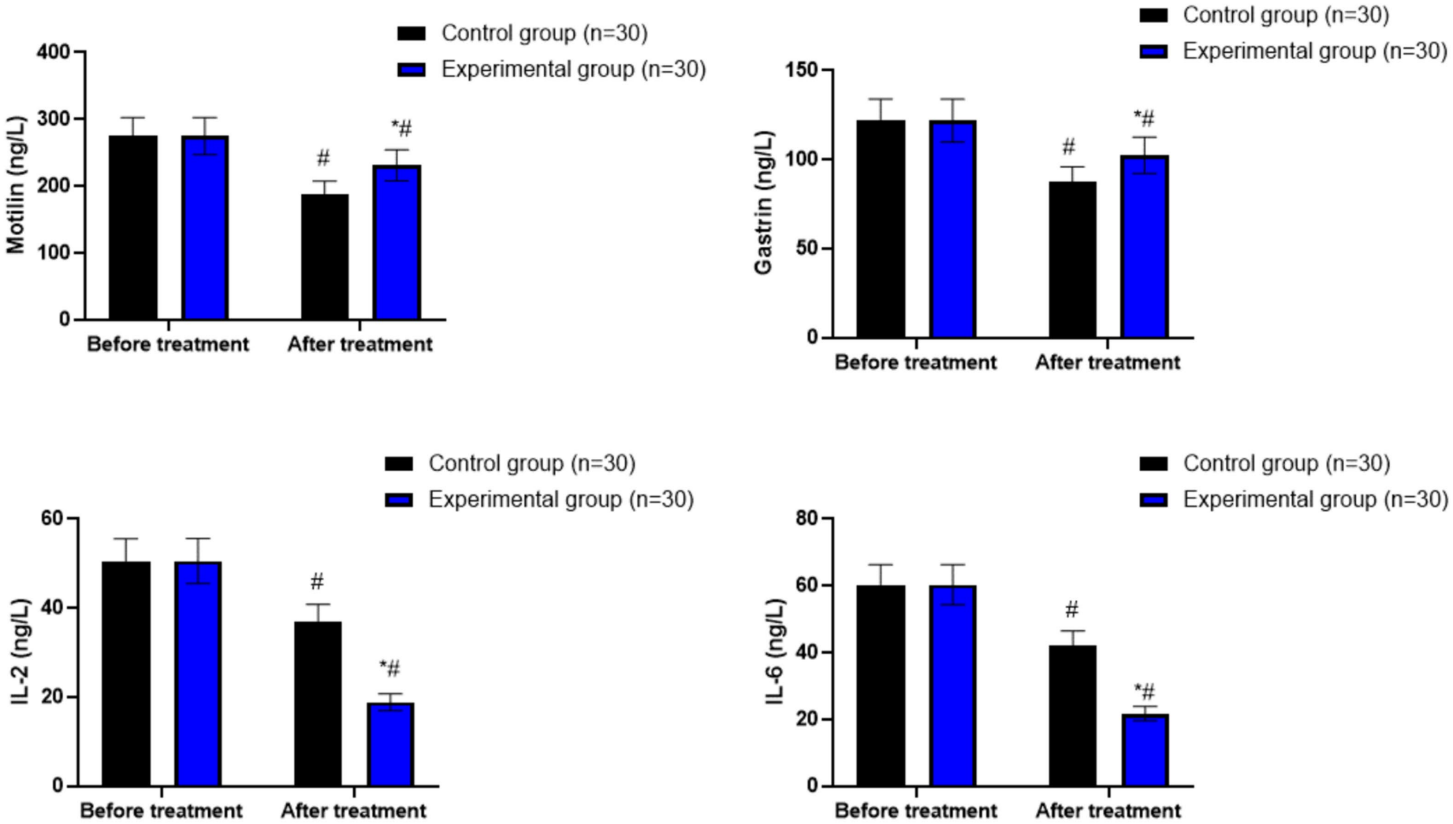
Laboratory indices in both groups. ^#^*p* < 0.05, compared with before treatment, ^*^p < 0.05, compared with the control group.

Before treatment, there were no significant differences in the serum levels of motilin, gastrin, IL-2, and IL-6 between the two groups (*p* > 0.05). After treatment, the serum levels of motilin, gastrin, IL-2, and IL-6 decreased significantly in both groups (*p* < 0.05, 95% CI: −30.08 to −12.38). Compared with the CG, the EG had significantly higher serum motilin and gastrin levels and significantly lower serum IL-2 and IL-6 levels (*p* < 0.05, 95% CI: 56.01–73.71; Figure 2).

### Physical comfort in both groups

**Figure 3:**
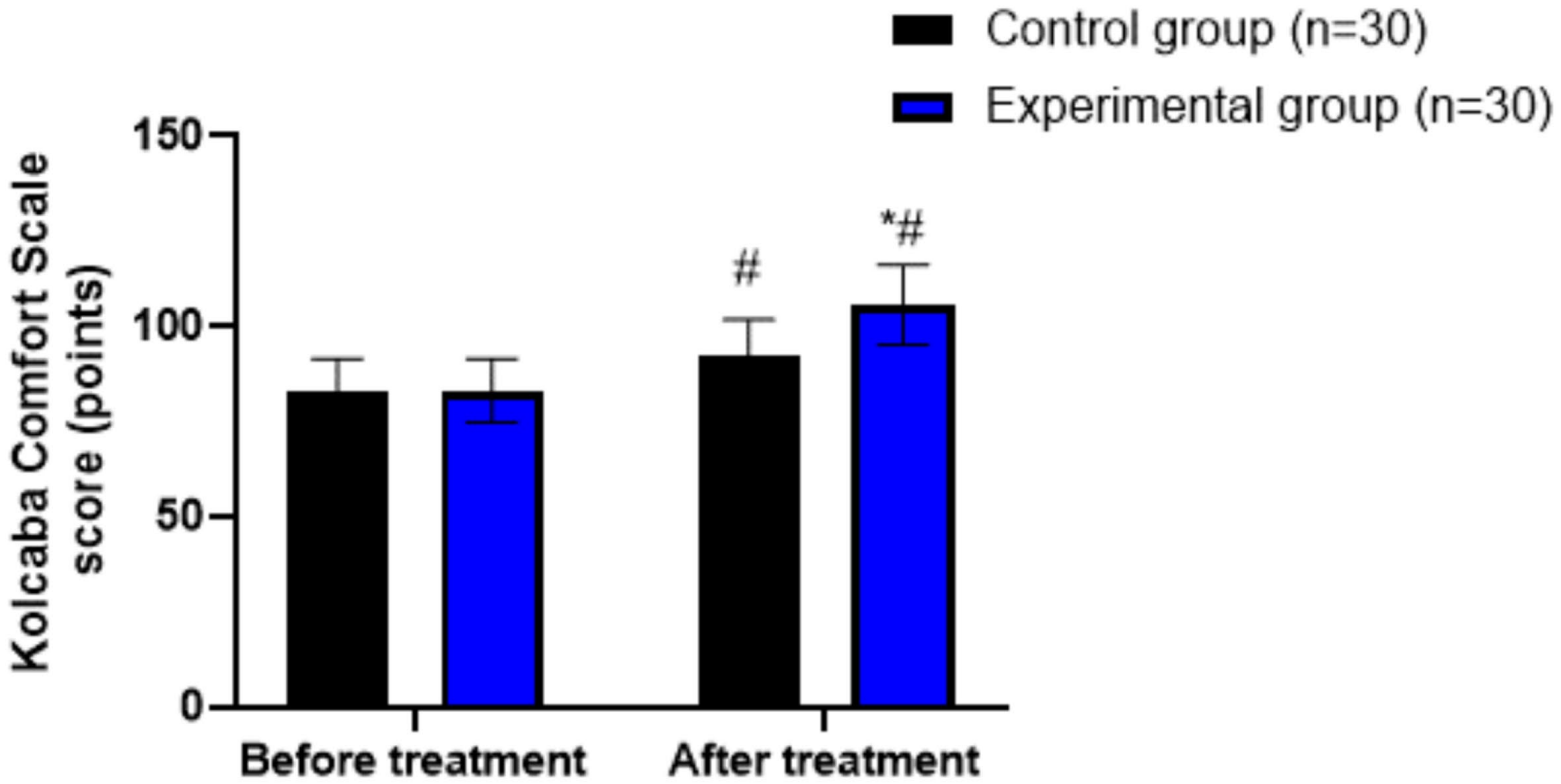
Physical comfort in both groups. ^#^*p* < 0.05, compared with before treatment, ^*^p < 0.05, compared with the control group.

Before treatment, there was no significant difference in the Kolcaba Comfort Scale score between the two groups (*p* > 0.05). After treatment, the Kolcaba Comfort Scale scores of both groups increased significantly (*p* < 0.05), and the EG score was significantly higher than that of the CG (*p* < 0.05) (Figure 3).

### Incidence of adverse reactions in both groups

As shown in Table 2, the incidence of adverse reactions in the EG was significantly lower than that in the CG after treatment (*p* = 0.037, 95% CI: 1.042–2.737).

**Table 2:** Incidence of adverse reactions between the two groups.

Groups	Cases	Nausea and vomiting	Abdominal distension	Total incidence rate
Control group	30	4	4	8 (26.67%)
Experimental group	30	1	1	2 (6.67%)

### Nutritive indices between the two groups

**Figure 4:**
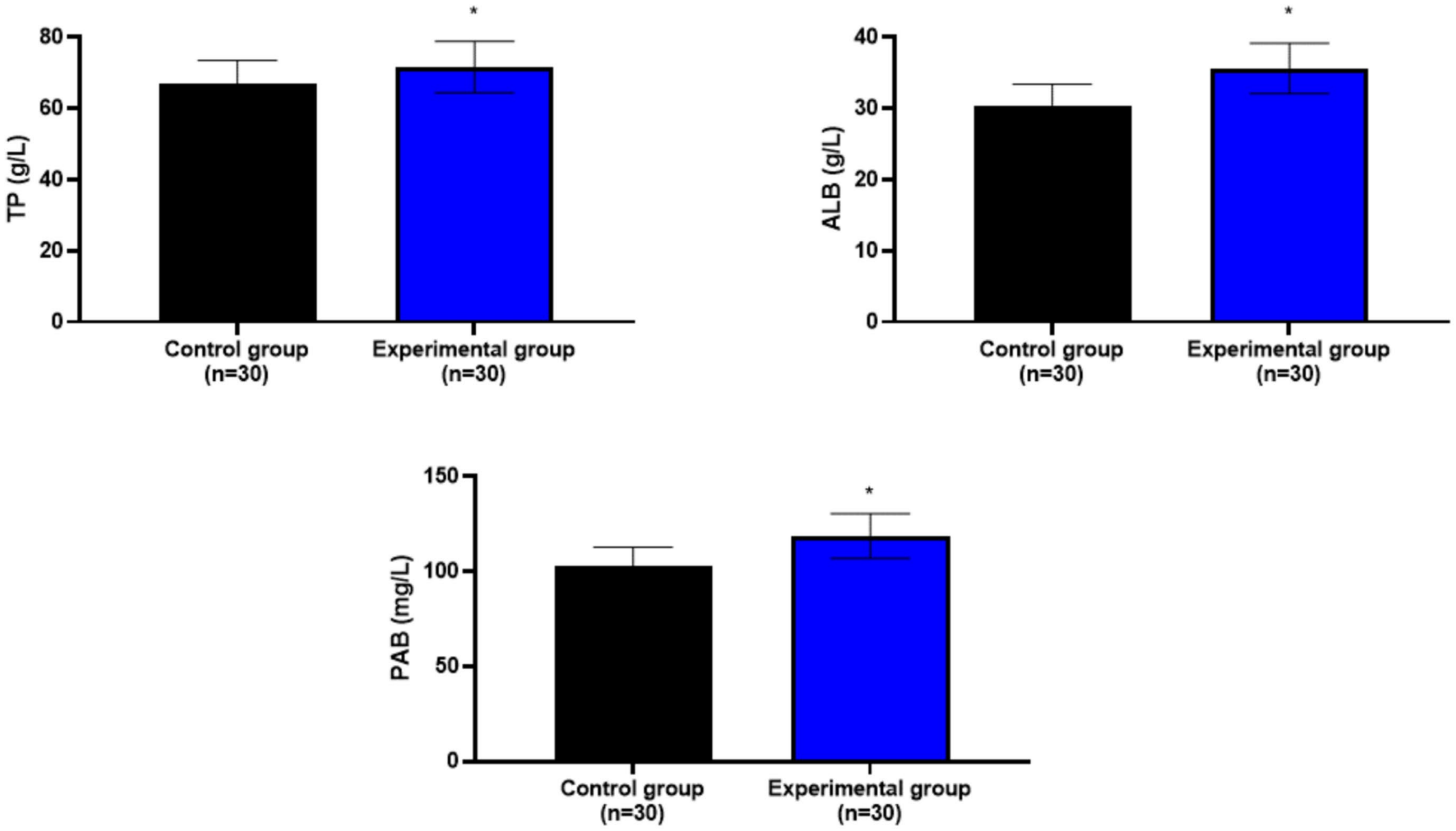
Nutritional indices in both groups. ^*^</sup < 0.05.

As shown in Figure 4, after treatment, the serum levels of TP, ALB, and PAB in the EG were significantly higher than those in the CG (*p* < 0.05).

## Discussion

As one of the primary treatment methods for advanced anteromedial knee osteoarthritis, UKA has also been shown to be effective in elderly patients. However, postoperative symptoms such as abdominal distension and constipation frequently occur, which seriously affect the patient’s quality of life (11). Postoperative gastrointestinal function is weakened, increasing the risk of malnutrition, electrolyte imbalance, and poor incision healing, which are not conducive to postoperative recovery (12).

Percutaneous acupoint electrical stimulation is a safe and effective non-pharmacological therapy that combines percutaneous nerve electrical stimulation and acupoint therapy (13). It delivers stimulation through electrodes placed on specific acupoints to exert therapeutic effects. Its electrode sheet features a single conductive embossment and connecting wire, enabling non-invasive, accurate, and convenient stimulation delivery (14).

From a modern physiological perspective, percutaneous acupoint electrical stimulation may regulate the efferent activity of the vagus nerve (15). The vagus nerve is a key component of the parasympathetic nervous system and plays a crucial role in regulating gastrointestinal function (16). Activation of the vagus nerve can stimulate the release of acetylcholine, a neurotransmitter that binds to muscarinic receptors on gastrointestinal smooth muscle cells, leading to smooth muscle contraction and thereby promoting gastrointestinal motility (17).

In addition to its effects on the vagus nerve, percutaneous acupoint electrical stimulation may also modulate cytokine levels, such as IL-2 and IL-6 (18). IL-2 is mainly produced by activated T cells and is involved in the regulation of immune responses (19); high levels of IL-2 can promote the proliferation and differentiation of T cells, potentially contributing to an exaggerated inflammatory response (20). IL-6, on the other hand, is a pro-inflammatory cytokine secreted by various cells (including macrophages) in response to injury or infection (21); excessive IL-6 production can lead to systemic inflammation and tissue damage (22). Studies have shown that percutaneous acupoint electrical stimulation can reduce the levels of IL-2 and IL-6 in patients, which may be related to its ability to improve microcirculation, relieve pain, and enhance immunity (23). By reducing the levels of these pro-inflammatory cytokines, percutaneous acupoint electrical stimulation can alleviate systemic inflammation, thereby improving gastrointestinal function (24). Inflammatory diseases are closely associated with immune cell polarization, and T cells and macrophages play important roles in the inflammatory process (25). Percutaneous acupoint electrical stimulation may promote the release of neurotransmitters, thereby reducing the production of pro-inflammatory cytokines and improving gastrointestinal motility (26).

Oral hydrolyzed protein products are mainly composed of hydrolyzed proteins obtained through protease hydrolysis (e.g., beef protein, muscle protein, and soy protein), consisting of short peptides and amino acids with a relative molecular weight ranging from 100 to 300 (27). Oral hydrolyzed protein is clinically used for the treatment of hypoproteinemia and can also be used to address nutritional deficiencies associated with systemic disorders such as burns, fractures, post-joint replacement, and poor wound healing, serving as an effective protein nutritional supplement (28).

Oral hydrolyzed protein contains 18 amino acids, 8 of which are essential amino acids, accounting for 56.1% of the total free amino acids (29). In addition, oral hydrolyzed protein is rich in short peptides and contains trace elements such as calcium, iron, and zinc (30). Short peptides can provide sufficient raw materials for protein synthesis, energy, and cell division, thereby improving the patient’s nutritional status and further enhancing their immune function (31).

While nutritional supplementation with oral hydrolyzed protein alone can improve the postoperative nutritional status of patients, it cannot fully explain the observed improvements in gastrointestinal function. Motilin and gastrin can promote gastrointestinal motility, regulate the transport of water and electrolytes in the gastrointestinal tract, stimulate phase III of myoelectric activity during the interdigestive period, promote forceful gastric contraction and small intestinal segmental movement, and accelerate intestinal peristalsis, thereby speeding up the passage of intestinal contents.

In our study, we found that the total effective rate of the EG was 86.67%, significantly higher than that of the CG (63.33%). Furthermore, the recovery time of bowel sounds, time to first flatus, and time to first defecation in the EG were significantly shorter than those in the CG. These results indicate that percutaneous acupoint electrical stimulation combined with nutritional support can improve gastrointestinal function and promote the recovery of patients after UKA, which is consistent with the findings of previous studies (32, 33).

This study confirmed that after treatment, the serum levels of motilin and gastrin in the EG were significantly higher than those in the CG. This suggests that percutaneous acupoint electrical stimulation combined with oral hydrolyzed protein can significantly improve the postoperative gastrointestinal function of patients, increase serum motilin and gastrin levels, accelerate the recovery of gastrointestinal function, and thereby support gastrointestinal function, which is consistent with relevant studies (34, 35).

Our study found that the serum IL-2 and IL-6 levels in the EG were significantly lower than those in the CG after treatment. Consistent with this, the existing literature has shown that percutaneous acupoint electrical stimulation can reduce surgical inflammation and perioperative stress responses in elderly patients undergoing knee joint surgery (36). Moreover, Ishikawa et al. (37) indicated that protein hydrolysate can alleviate postoperative inflammation.

After treatment, the EG had a significantly higher Kolcaba Comfort Scale score, a significantly lower incidence of adverse reactions, and significantly higher serum levels of TP, ALB, and PAB compared with the CG. These data suggest that percutaneous acupoint electrical stimulation combined with nutritional support can improve the physical comfort of patients after UKA, reduce the incidence of adverse reactions, and enhance their nutritional status, which is consistent with previous studies (38, 39).

This study has several limitations. First, the use of the Kolcaba Comfort Scale in this specific postoperative orthopedic context may have potential limitations. The scale may not be sufficiently sensitive to capture unique comfort issues in postoperative orthopedic patients, such as specific types of joint-related pain, and may not fully account for cultural and individual differences in comfort perception. Second, multiple statistical comparisons were conducted without adjustment for type I error, which may increase the risk of false-positive findings. Therefore, the results should be interpreted with caution, and future studies should incorporate corrections such as the Bonferroni method to enhance statistical rigor. Third, due to the nature of the intervention, patients were not blinded to group assignment, which may introduce performance bias. Although outcome assessors and statisticians were blinded, the lack of patient blinding remains a limitation that could influence patient-reported outcomes such as comfort scores. Therefore, while the combined treatment shows promising results, further research with larger sample sizes, more rigorous statistical methods, and in-depth exploration of clinical endpoints is needed to fully determine its clinical value and applicability.

## Conclusion

This study demonstrates that percutaneous acupoint electrical stimulation combined with nutritional support exerts a positive effect on promoting the recovery of gastrointestinal function and improving the nutritional status of patients after UKA.

## Data Availability

The original contributions presented in the study are included in the article/supplementary material, further inquiries can be directed to the corresponding author.
